# Different Oral Antithrombotic Therapy for the Treatment of Ventricular Thrombus: An Observational Study from 2010 to 2019

**DOI:** 10.1155/2022/7400860

**Published:** 2022-02-24

**Authors:** Qing Yang, Xinyue Lang, Xin Quan, Zebin Gong, Yan Liang

**Affiliations:** ^1^Fuwai Hospital, Chinese Academy of Medical Sciences & Peking Union Medical College, National Center for Cardiovascular Diseases, No. 167 Beilishi Road, Xicheng District, Beijing 100037, China; ^2^Medical Research & Biometrics Center, National Center for Cardiovascular Diseases, Chinese Academy of Medical Sciences, Beijing 102300, China

## Abstract

**Methods:**

This retrospective observational study was conducted from 2010 to 2019 in National Center of Cardiovascular Diseases of China. We included patients with VT confirmed by imaging. The primary outcome was the rate of thrombus resolution. Hazard ratio (HR) was calculated with or without adjustment for covariates using Cox proportional hazards regression models.

**Results:**

463 patients were included. 43.0% received VKAs, 16.6% received NOACs, and 40.4% received APT. Over a median of 468 days' follow-up, NOACs group was more likely to have the thrombus resolved within 12 months' follow-up than VKAs (HR 2.28, 95% CI 1.57 to 3.31) or APT (HR 2.92, 95% CI 1.97 to 4.33). After adjustment for baseline variables, the significance remained in the comparison of NOACs versus VKAs (HR 2.13, 95% CI 1.41 to 3.22) as well as NOACs versus APT (HR 2.55, 95% CI 1.53 to 4.27). No significant differences were identified in bleeding rate, thromboembolism rate, or all-cause death in 12 months' follow-up.

**Conclusion:**

Our findings showed that patients who were male, diagnosed with MI with or without ventricular aneurysm, or diagnosed with coronary artery diseases medical history had a risk of thrombus unresolved. Patients with NOACs had a higher resolution and a similar safety profile comparing VKAs or APT, which persisted after adjusting for other factors. Large randomized controlled trials are required urgently. This trial is registered with NCT05006677.

## 1. Introduction

The incidence of ventricular thrombus (VT) in myocardial infarction (MI) has been reduced from 20%–60% to 2%–5% with the advance of antithrombotic therapy [1, 2]. Based on myocardial infarction or stroke guidelines, warfarin is typically used for anticoagulation. International guidelines recommended that using warfarin anticoagulant therapy for ST elevation myocardial infarction (STEMI) patients with asymptomatic left VT was reasonable *(Class II a, Level C evidence)* [3, 4]. Recently, non-vitamin K antagonist oral anticoagulants (NOACs), dabigatran etexilate, rivaroxaban, apixaban, and edoxaban, have made impressive results in the anticoagulating treatment of nonvalvular atrial fibrillation and venous thrombotic diseases [5, 6]. Studies showed that the rate of thrombus resolution ranged from 83% to 100% with a low incidence of stroke or systemic embolism in patients with left VT who received NOACs [7, 8]. Owing to its special features including rapid onset of action or no frequent monitoring, NOACs indicated a better effectiveness and safety than warfarin [9]. In the stroke and transient ischemic attack (TIA) prevention guidelines, for patients with MI combined with ischemic stroke or TIA, the treatment with dabigatran, rivaroxaban, or apixaban for 3 months could be regarded as an alternative treatment to VKAs to prevent recurrence stroke or TIA *(Class II b, Level C evidence)* [10]. 2017 European guidelines also recommended that STEMI patients with left VT should maintain anticoagulation therapy for up to 6 months under the guidance of repeated imaging (*Class II a, level C evidence*) [11]. Up to date, quite a few studies reported that NOACs had comparable safety and efficacy outcomes in the treatment of VT [12–24]. Michael et al. (2021) conducted a meta-analysis to compare NOACs to VKAs for the treatment of left ventricular thrombus, including a total of 18 studies with 2666 patients, and their result showed that NOACs had less 37% risk of stroke than VKAs, while no significances were observed in thrombus resolution, systemic embolism, bleeding, or all-cause death events. Some researchers reported that NOACs had fewer bleeding events than VKAs; Kido et al. [25] and Jones et al. [26] found that the use of NOAC was significantly associated with lower bleeding event rates. Except the two agents for anticoagulation in VT, most patients with coronary artery diseases (CAD) were administered the antiplatelet therapy (APT) such as aspirin, clopidogrel, or ticagrelor to treat VT. However, it remains unknown which antithrombotic therapy in patients with VT is more beneficial. We aimed to evaluate the utilization of oral antithrombotic agents in VT patients and to analyze factors for outcomes, providing a potential resource to explore more evidence on the comparison of the efficacy and safety among different antithrombotic therapies.

## 2. Methods

### 2.1. Patient Population

This retrospective observational study was conducted from July 2010 through October 2019 using electronic medical records of Fuwai Hospital, National Center of Cardiovascular Diseases in China, which was registered in ClinicalTrials.gov: NCT 05006677. We included patients who had a diagnosis of VT when discharged. The including criteria were as follows: (1) Age should be over eighteen years, regardless of sex or occupation. (2) Patients diagnosed as VT received a newly prescribed NOAC, VKAs, or APT within four weeks of first diagnosis. The dosing of NOAC depended on patient-specific factors (e.g., creatinine clearance), and the warfarin dose was titrated to maintain an internationally standardized ratio (INR) goal of 2.0 to 3.0. (3) The VT should be found newly within three months, for old thrombi were more likely to be mechanized or calcified to resolve. Patients who had received more than four weeks' standard antithrombotic therapy (e.g., aspirin 100 mg/d combined with a P2Y12 inhibitor, clopidogrel 75 mg/d or ticagrelor 90 mg twice daily, rivaroxaban 15 mg twice daily) or switched agents during the treatment period were excluded. All medications including anticoagulant drugs were based on the preference of patients, following counselling with their physician.

### 2.2. Definitions

The diagnosis of VT was confirmed by transthoracic echocardiography or transesophageal echocardiography, contrast-enhanced echocardiography, computed tomography, or cardiac magnetic resonance imaging. When these imaging tools were not consistent, we would review the image and reach a conclusion. VT was defined as an abnormal echo mass in the ventricular cavity, whose edge was different from the ventricular endocardium. The shape of VT could be variable and was mostly circular bulge, which was distinguished from the internal structures such as papillary muscles, tendon, or trabeculations [27]. Multiple sections confirmed the existence of thrombus.

### 2.3. Outcomes

The primary outcome was the rate of thrombus resolution determined by repeat imaging, and the secondary outcomes included thromboembolism events, bleeding, and all-cause death during the period of follow-up. Thromboembolism events were defined as the composite of ischemic stroke or transient ischemic attack, acute coronary emboli (including myocardial infarction), acute pulmonary embolism, or acute peripheral artery emboli (limb, renal, or digestive arteries). Bleeding events were defined as International Society on Thrombosis and Haemostasis (ISTH) major bleed [28], clinically relevant nonmajor bleed [29], or minimal bleed not fulfilling criteria for the above two types of bleeding.

### 2.4. Follow-Up

**Figure 1:**
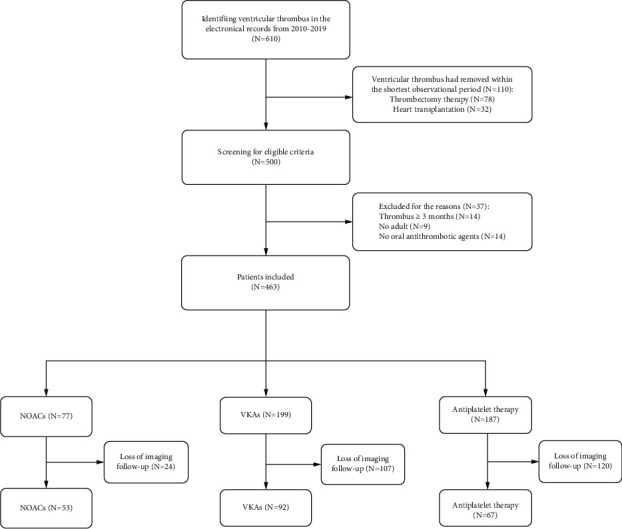
Flow diagram to show patient inclusion and exclusion criteria. 610 patients were found with VT and 463 were included in our analysis: 199 received VKAs, 77 received NOACs, and 187 received antiplatelet therapy. A total of 212 patients had the follow-up image: 53 in NOACs, 92 in VKAs, and 67 in antiplatelet therapy. VT: ventricular thrombus; *N*: number of patients; NOACs: non-vitamin K antagonist oral anticoagulants; VKAs: vitamin K antagonists.

The follow-up time was from the date of first diagnosis of VT to the date of complete resolution of VT or last documented medical records or until death within 12 months. Data were collected through electronic medical records and surveys at baseline (soon after VT diagnosis), 6 weeks (range: 4 to 8 weeks), 12 weeks (range: 10 to 14 weeks), 6 months (range: 22 to 26 weeks), and 12 months (range: 46 to 50 weeks). Two colleagues (Q.Y. and X.Q.) made a survey via phone interviews with the patients or their relatives, obtaining the clinical events or image reports, and confirmed the image or events by inquiring for the local hospital medical records or the attending physician. Details are in Figure 1.

### 2.5. Data Collection and Analysis

The number of patients discharged and the number of patients with a diagnosis of VT from 2010 to 2019 were collected in the electronic system of Fuwai Hospital. Two colleagues (Z.G. and X.L.) calculated the proportion of VT in Fuwai Hospital separately and made a consistent agreement. A data collection form was conducted to extract the patients' characteristics, including demographic characteristics, presenting diagnosis, previous medical history, laboratory data, cardiac imaging data, and antithrombotic therapy. Two colleagues (Q.Y. and X.Q.) extracted the data independently and compared the results to ensure coherence, and an additional scholar resolved the discrepancies. Data were collected from electronic medical records and written consent was waived owing to minimal patient risk. Oral consent was obtained at the time of the telephone interview.

### 2.6. Statistics Analysis

Normally distributed continuous data were presented as mean and standard deviation (SD) and nonnormally distributed continuous data by median and interquartile range (IQR), and the dichotomous data were calculated by the frequency and percentage [30]. Analysis of variance was used to compare normally continuous variables and Kruskal–Wallis H test was used to compare nonnormally distributed continuous variables. The Pearson chi-squared test or Fisher's exact test (when more than 20% of cells have expected frequencies <5) was used to compare categorical data. Hazard ratio (HR) was calculated with or without adjustment for covariates using Cox proportional hazards regression models. The thrombus resolution was entered as the outcome in the Cox regression analysis. The Kaplan–Meier method was used to calculate the cumulative event probability of antithrombotic agents and the time was coded in days from the date of first diagnosis of VT to the date of complete resolution of VT in patients with VT. Log-rank test was used to compare the cumulative event among groups. All comparisons were considered as two-sided, and *p* < 0.05 was considered as statistical significance. Incidence rate estimates were calculated in Excel 2016. All other analyses were scheduled for completion with R Studio and R, version 3.5.1 (R Foundation for Statistical Computing).

## 3. Results

### 3.1. Proportion of Ventricular Thrombus

**Figure 2:**
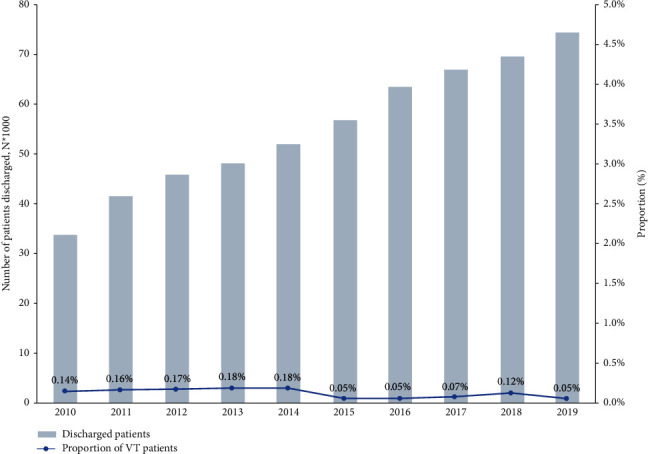
The number of discharged patients (left axes, blue column) and the proportion of ventricular thrombus patients (right axes, line chart).

Figure 2 depicts the number of discharged patients and the proportion of VT patients accounted by year in Fuwai Hospital from June 2010 to June 2019. With a total of 552,943 patients in discharge, VT was found in 610 patients (0.11%). The median rate of VT was 0.13% and there was a trend of decline of VT in recent years, ranging from 0.18% to 0.05%. See Table S1 in the electronic Supplementary Materials for details.

### 3.2. Patients Characteristics

Among 610 patients, 78 individuals received the thrombectomy therapy or ventricular aneurysm resection within 6 weeks since the VT was found in the first time, and 32 received the heart transplantation within 6 weeks; 14 patients had a long history of VT more than three months; 9 patients were below eighteen years of age; 14 patients received no oral antithrombotic agents.

We included 463 eligible patients: 43.0% (*n* = 199) of them received VKAs, 16.6% (*n* = 77) received NOACs, and 40.4% (*n* = 187) received APT. In patients with NOACs, most of them were given rivaroxaban (*n* = 72, 93.5%) and four patients were administered with dabigatran, while apixaban was used for only one patient. The mean age of participants varied among each group, and patients in the NOACs group had the youngest age with a median age of 45 years. More than 60% of patients were male among each group. The presenting diagnosis, ischemic cardiomyopathy, ventricular aneurysm, dilated cardiomyopathy, hypertrophic cardiomyopathy, and other diseases, differed in the three groups when VT was first found in patients (*p* < 0.0001). The baseline left ventricular ejection fraction (LVEF) also showed a significant difference, while D-Dimers were similar among groups. Patients with acute MI received parental anticoagulants, heparin, low-molecular heparins, or fondaparinux, before urgent perfusion therapy, followed by oral anticoagulation with an NOAC or VKA. The median of administering parental anticoagulants was 6 (IQR 3–10) days, and there was a significant difference among NOACs, VKAs, and APT group (*p* < 0.0001). Based on the data from imaging examination, most of the VT was located in left VT, while the number and the length or thickness of VT were nonsignificant among groups (*p* = 0.6361, *p* = 0.6056, separately) (Table 1).

Within the entire study population, the investigators could track survival and health status of these 463 patients within the period of follow-up. 212 (NOACs = 57 versus VKAs = 92 versus AP = 67) patients had the imaging follow-up, while a total of 251 patients were lost to the image follow-up: 246 patients had the phone review without the image reports, and five other patients were dead not until the first follow-up visit at 6 weeks. We performed a comparative analysis to compare the demographic and baseline characteristics between those who completed the follow-up and those who were lost to imaging follow-up (*N* = 212 versus *N* = 251), which showed no significant difference (Table S2). Analyzing the characteristics among the three group with imaging follow-up, these yielded results similar to those in the total population (Table S3).

### 3.3. Primary Outcome

#### 3.3.1. Unadjusted Thrombus Resolution

**Figure 3:**
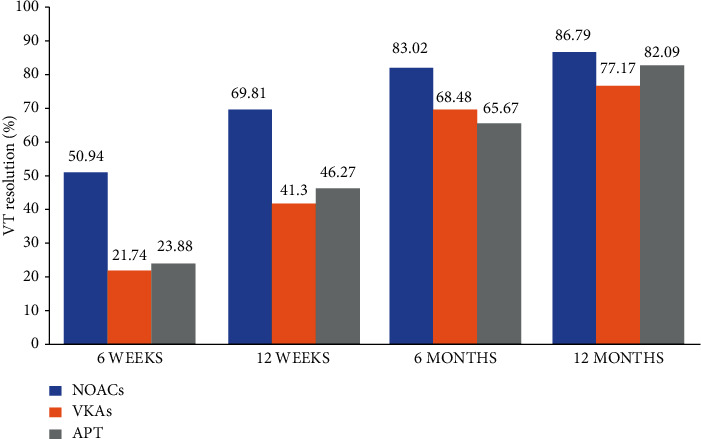
Bar chart comparing rates of thrombus resolution in patients with different antithrombotic therapy. A significant unadjusted difference in rates of resolution was observed among patients on NOACs, VKAs, and APT at 6 weeks' (*p* = 0.0005), 12 weeks' (*p* = 0.0032), and 6 months' (*p* = 0.0844) follow-up, while there was no difference at 12 months' (*p* = 0.3515) follow-up. VT: ventricular thrombus; NOACs: non-vitamin K antagonist oral anticoagulants; VKAs: vitamin K antagonists; APT: antiplatelet therapy.

The median duration of imaging follow-up was 41 (32–48) days in 6 weeks, 77 (67–90) days in 12 weeks, 128 (112–150) days in 6 months, and 270 (227–330) days in 12 months. At 12 months' follow-up, 212 patients had a follow-up imaging data, while 172 (81.13%) patients of them had confirmed resolution of their thrombus (Table 2). During the whole period of follow-up, no patients were found to switch the anticoagulant treatment.  Figure 3 shows that, at 6 weeks' follow-up, 27 (50.94%) patients had a resolution of thrombus in the NOACs group, and 20 (21.74%) patients and 16 (23.88%) patients separately had the VT disappearing in the VKAs and APT groups (*p* = 0.0005). At 12 months' follow-up, thrombus completely resolved in 46 of 53 (86.79%) patients in the NOACs group, in 71 of 92 (77.17%) patients in the VKAs group, and in 55 of 67 (82.09%) patients in the APT group without significant difference among groups (*p* = 0.3515).

#### 3.3.2. Adjusted Thrombus Resolution

**Figure 4:**
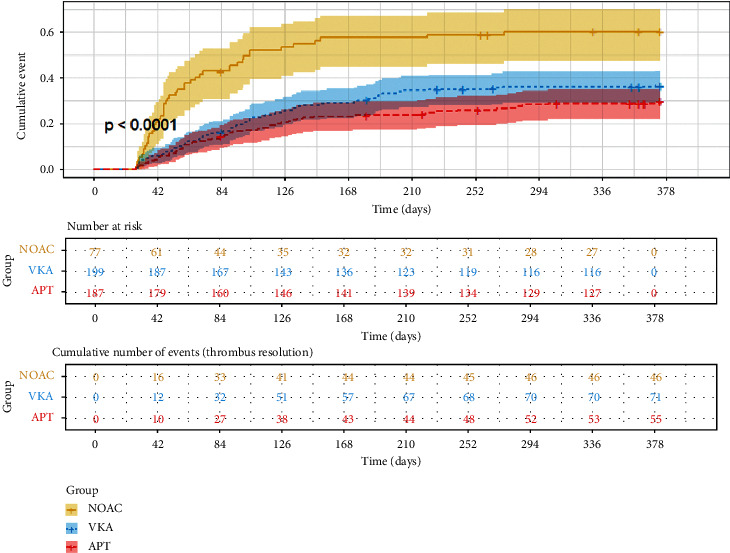
Cumulative event probability curve for thrombus resolution of NOACs, VKAs, and APT. Kaplan–Meier method was used to calculate the cumulative event probability of three antithrombotic agents and the time was coded in days from the date of first diagnosis of VT to the date of complete resolution of VT in patients with VT. Log-rank test was used to compare the cumulative event among groups (*p* < 0.0001). VT: ventricular thrombus; NOACs: non-vitamin K antagonist oral anticoagulants; VKAs: vitamin K antagonists; APT: antiplatelet therapy.

Comparing VKAs and APT, NOACs were more likely to have the thrombus resolution within 12 months' follow-up (HR 2.28, 95% CI 1.57 to 3.31, *p* < 0.001; HR 2.92, 95% CI 1.97 to 4.33, *p* < 0.001, respectively). No significance was observed between VKAs and APT in thrombus resolution. In the multivariable analysis, adjusted for clinically significant factors on the basis of biological knowledge (e.g., presenting diagnosis, history of CAD and heart failure, LVEF, and D-Dimer) combining these significant variables in univariable analysis, there was still a significance in the comparison of NOACs versus VKAs as well as NOACs versus APT (HR 2.13, 95% CI 1.41 to 3.22, *p* < 0.001; HR 2.55, 95% CI 1.53 to 4.27, *p* < 0.001, respectively) (Table 3). The cumulative event probability curve showed that the thrombus resolution among patients with different antithrombotic agents differed, and obviously the NOACs group had a better resolution than the VKAs and APT group (log-rank test, *p* < 0.0001) (Figure 4).

### 3.4. Secondary Outcomes

#### 3.4.1. Bleeding

Over a median follow-up of 465 (125–1216) days, bleeding occurred in a total of 25 (5.4%) patients: one patient (1.3%) in the NOACs group, 12 patients (6.0%) in the VKAs, and 12 patients (6.4%) in the APT group (Table 4 and Table S4). No significant difference was observed at 12 months' follow-up in bleeding rate among the NOACs, VKAs, and APT groups (*p* = 0.216). Five (2.5%) bleeding events and eight (4.3%) bleeding events during the initial hospitalization occurred in the VKAs group and APT group, respectively. Major bleeding occurred in two (1.1%) patients in the APT group (one in gastrointestinal and one in cerebral) and six other patients had minor bleeding (e.g., skin, nose). None of the patients were transfused at the time of bleeding.

#### 3.4.2. Thromboembolism

We observed no significant difference in thromboembolism rate among the NOACs, VKAs, and APT groups (0%, 1%, and 1%, *p* = 1.000) at 12 months' follow-up (Table 4). One (0.5%) patient had a cerebral embolism in the APT group and one (0.5%) patient had an embolism in the lower limbs in the VKAs group. No thromboembolism events were recorded in the NOACs group.

#### 3.4.3. All-Cause Death

Seven (1.5%) patients died: five (2.5%) in the VKAs group and two (1.1%) in the APT group (Table 4 and Table S4). Two (1.0%) patients in the VKAs group died from severe heart failure or other dreadful infectious diseases (one was dead at 24 weeks with thrombus resolution and the other man was dead at 45 weeks after hospital discharge without thrombus resolution) and the other three (1.5%) patients in the VKAs group died during the initial hospitalization. Two (1.1%) patients in the APT group died due to the aggravating multiorgan diseases, occurring within 6 weeks' follow-up. No significant difference was observed in all-cause death among the NOACs, VKAs, and APT groups at 12 months' follow-up (*p* = 0.392).

### 3.5. Further Analysis

To further assess the relation between thrombus resolution rates and the factors of patients, we conducted a Cox proportional hazards regression. In the univariable analysis, males were less likely to have the resolution of thrombus, with the HR for women being 0.69 (95% CI 0.48 to 0.98, *p* = 0.036). Patients who were diagnosed as MI with ventricular aneurysm had a greater risk to have the thrombus unresolved than those who were diagnosed with dilated cardiomyopathy or “other” diseases (HR 0.49, 95% CI 0.32 to 0.75, *p* < 0.001; HR 0.47, 95% CI 0.3 to 0.73, *p* < 0.001, respectively). When patients had a history of CAD, the thrombus resolution could be decreased by 0.6% (HR 0.61, 95% CI 0.45 to 0.82, *p* = 0.001); by contrast, for those with heart failure, they might be more likely to have a thrombus resolution (HR 1.5, 95% CI 1.11 to 2.02, *p* = 0.008). With LVEF increasing by 1%, the possibility of resolution decreased slightly by 1% (HR 0.99, 95% CI 0.98 to 0.999, *p* = 0.032). Similarly, with D-Dimer elevating 1 ug/mL, the resolution decreased by 0.9% (HR 0.94, 95% CI 0.88 to 0.999, *p* = 0.047) (Table 3 and Table S5).

## 4. Discussion

### 4.1. Key Findings and Clinical Implication

In our study, we have the following findings: (a) NOACs appeared to be a reasonable and more convenient option for patients with VT, compared with warfarin, especially rivaroxaban. (b) NOACs, VKAs, and APT had similar safety profiles in bleeding, thromboembolism, and all-cause death within 12 months. (c) Being male, presenting diagnosis with MI with or without ventricular aneurysm, and medical history of CAD were factors to have a risk of thrombus unresolved. More prospective trials are critical to further provide strong evidence on this topic.

### 4.2. Non-Vitamin K Antagonist Oral Anticoagulants in Patients with Ventricular Thrombus

Obviously, NOACs have several advantages, stable and predictable anticoagulant effect as well as no food or less drug interaction, no restriction of treatment window, and so on [9, 31], which might have a better safety profile than VKAs [7, 21]. One of the main advantages of promoting the use of NOACs is that the treatment of NOAC can be performed at a fixed dose without monitoring routine laboratory frequently, except for those special populations as the elder or renal dysfunction [32, 33]. Moreover, as we all know, the biomarkers of fibrinolysis or thrombogenesis (e.g., D-Dimer, prothrombin time, activated partial thromboplastin time, and Anti-Xa activity) could test the concentration of NOACs [34, 35], and, interestingly, Miyazawaa et al. [36] found that the biomarkers of inflammation (e.g., high-sensitivity C-reactive protein, hs-CRP) were related to left atrial thrombus resolution significantly when patients were administered with rivaroxaban prospectively, and the higher biomarker at baseline, the more chance of the thrombus being resolved. Mechanisms like endothelial damage, increased platelet activation, and increased expression of fibrinogen could be possibly accounted for it. Therefore, for patients with VT, they can benefit from NOACs in a more convenient and accurate mode, and for our clinicians we can utilize those variations of biomarkers to monitor the effect or safety of anticoagulants or guide their treatment and predict the prognosis of patients.

In aspects of our key findings, patients who received NOACs had a greater resolution more than 1 time than those with VKAs or APT, which was consistent with several articles [12, 26]. Researchers have explored the effectiveness of NOACs in the treatment of VT. Sedhom et al. collected 85 cases of direct oral anticoagulants for treatment of left VT. 74 patients (87%) were prescribed an NOAC and 93.2% of them had a complete resolution (69/74) [37]. Our team summarized the studies on the comparison of NOACs, VKAs, and APT.  Table S6 showed the result of literature reviews. Most of studies claimed that NOACs were comparable with or even outweighed VKAs. In a retrospective study including 101 patients with left VT, 41 (40.6%) received NOACs and 60 (59.4%) received VKA at 1-year follow-up, and the NOACs group had a faster and earlier thrombolysis rate than VKA (82% versus 64.4%), a similar rate of thromboembolism (5% versus 2.4%, *p* = 0.388), and a lower incidence of bleeding events (0% versus 6.7%, *p* = 0.03) [26]. Another study also found that the time of thrombus resolution in the NOACs group was faster than that in the VKAs group (63 days [IQR 40, 138] versus 123 days [IQR 86, 244], *p* = 0.003) [12]. Bass et al. 2021 retrospectively included 949 patients diagnosed with left VT; 180 (19%) received NOACs and 769 (81%) received warfarin, and no differences in new embolism or bleeding events were observed (NOACs versus warfarin: 7.8% versus 11.7%, *p* = 0.13; 10.9% versus 7.8%, *p* = 0.40) [38].

Several meta-analyses reported results similar to ours. Burmeister et al. (2021 concluded) that patients with NOACs showed a higher rate of thrombus regression (risk ratio = 1.18, 95% confidence interval 1.04–1.35, *p* = 0.01) while there was no significant difference between the NOACs and VKA groups in stroke, thromboembolism, or any bleeding event [24]. Two reviews, Saleiro et al. [18] and Camilli et al. [39], comparing NOACs and VKAs in patients with left VT have been published; while the former paper suggested that NOACs were as effective as VKAs for VT with a similar risk of thromboembolism/stroke and bleeding, the latter paper concluded that NOACs were comparable to VKA in terms of thrombus resolution, providing lower bleeding rates and an increase in thromboembolism events in the NOACs group. As regards thromboembolism events, our study found that thrombosis possibly occurred in hospital in patients who were already taking anticoagulants (e.g., patients who had acute coronary diseases or planning surgery had received parental anticoagulation). From our perspective, there could be two reasons for the situation. First those patients who were given an earlier anticoagulation had a quite severe cardiac dysfunction or were in the unstable situation, both of which could cause inflammation, release a series of thrombotic factors, and form a state of hypercoagulability, resulting in the ventricular thrombus. Second, the short duration or small dosage of anticoagulation could produce low efficacy of anticoagulation, and some patients who were already on oral anticoagulation might have a poor adherence such as bad monitoring of INR or irregular treatment.

Otherwise, while most studies mainly compared the efficacy and safety between NOACs and VKAs [12–23] (Table S6), our study firstly offered a new idea towards exploring APT in VT treatment. Our study showed that the rates of bleeding, embolism, and all-cause death were similar among the NOACs, VKAs, and APT groups within a 12 months' follow-up. Our subgroup analysis showed that most of patients in the NOACs group had a great thrombus resolution (>80%) and an earlier resolution (50.94% at 6 weeks' follow-up, *p* = 0.0005) with low prevalence of events of bleeding (<1%), embolism (<1%), and all-cause death (<1%). Table S4 shows that patients with rivaroxaban had a high resolution of VT (89.6%) and a low rate of bleeding (1.4%) as well as embolism and all-cause death events (0%, 0%), compared to those who received warfarin or dual APT. Due to the complexity of dosing of these agents, we failed to assess the exact effectiveness and needed more trials to make a supplementary. In our result, patients who received only APT without anticoagulation therapy had an 82.09% resolution, while the comparison with other groups was nonsignificant. According to the study population, patients with APT in whom cardiac ischemic events happened were all diagnosed with ischemic cardiomyopathy or combined with ventricular aneurysm, which might reflect that the thrombus possibly occurred together with this MI and thus was more likely easier to resolve. In our result, patients administered APT had a better systolic cardiac function and lower D-Dimer than the patients in the NOACs and VKAs groups, and all of them had only one thrombus, which indicated a greater possibility to have the thrombus resolved. More reliable evidence was needed to illustrate the mechanism. Whether one or dual APT with or without anticoagulants was more effective or safe was unknown. Galli et al. [40] found that, with APT, NOACs could inhibit factor Xa to modulate thrombin generation, providing effective antithrombotic effects. Take rivaroxaban for example; it significantly reduces thrombin production and is associated with the trend of TF induced decrease in platelet aggregation. That is to say, combining rivaroxaban with APT reduces thrombin generation. Since the number of patients with antiplatelet agent combining NOACs and VKAs was quite small, we had no further conclusion to give more evidence and more studies are required to explore the efficacy and safety of NOACs combined with APT for the treatment of VT.

### 4.3. Limitations

There were several limitations in our paper. First, it was a retrospective observational study with the inherence of selective bias, and the sample was small to explore more strong evidence. There was a lack of forward data of INR; we could underestimate bleeding in patients with warfarin who had a poor control. Second, owing to the various individual conditions, we lacked sufficient power to assess the optimal type or dosing of different antithrombotic agents for patients with VT. There were a few patients we included who were already taking anticoagulants, which might impact on the outcomes, even though the period was within one month. Finally, it was unavoidable that some possible confounding factors existed in the research according to different previous diseases or combined condition, or complex treatments, and we failed to find out all confounding factors which were significantly associated with the thrombus resolution, even though we performed univariable Cox proportional hazards regression.

To date, the therapeutic effect of antithrombotic agents on VT has not been formally evaluated in randomized controlled trials, which may be associated with the small number of patient cases and difficulty in enrollment. In light of the 2016 CHEST practice guidelines for the treatment of venous thromboembolism, an updated version may expand the use of loading doses of NOACs for the treatment of intracardiac thrombosis [11]. Four randomized trials (NOACs versus VKAs) are currently in the pilot phase, aiming to evaluate the efficacy of rivaroxaban versus warfarin (EARLY-MYO-LVT trial) [41] and dabigatran versus warfarin (NCT 03415386) for the treatment of left VT, respectively. Two other ongoing prospective clinical trials randomly assign patients with left VT to receive the treatment of either warfarin or apixaban (NCT03232398 and NCT02982590).

## 5. Conclusion

Our findings showed that patients who were male, diagnosed with MI with or without ventricular aneurysm, or diagnosed with CAD medical history had a risk of thrombus unresolved. Comparing VKAs and APT, patients with NOACs had a higher resolution and a similar safety profile, while there were no significant differences in the rates of thromboembolism, bleeding events, or all-cause death. Large randomized controlled trials are urgently required to further evaluate the effectiveness and safety of different antithrombotic therapies in patients with VT.

## Figures and Tables

**Table 1:** Baseline characteristics of patients with ventricular thrombus (*N* (%)).

	NOACs (*N* = 77)	VKAs (*N* = 199)	Antiplatelet therapy (*N* = 187)	*p* value
Age, y (mean ± SD)	45.3 ± 17.2	49.3 ± 15.1	57.4 ± 12.3	<0.0001
Male	55 (71.4)	160 (80.4)	160 (85.6)	0.028
BMI, kg/m^2^ (median (IQR))	23.9 (22.4–26.9)	23.8 (21.3–26.7)	24.7 (22.6–26.6)	0.385
Presenting diagnosis				<0.0001
ICM	11 (14.3)	53 (26.6)	74 (39.6)	—
Ventricular aneurysm	15 (19.5)	44 (22.1)	105 (56.1)	—
DCM	19 (24.7)	57 (28.6)	5 (2.7)	—
HCM	2 (2.6)	8 (4.0)		—
Others^†^	30 (39.0)	37 (18.6)	3 (1.6)	—
Prior medical history				
Coronary artery disease	30 (39.0)	101 (50.8)	171 (91.4)	<0.0001
Atrial fibrillation	6 (7.8)	27 (13.6)	10 (5.3)	0.018
Heart failure	46 (59.7)	114 (57.3)	33 (17.6)	<0.0001
Hypertension	23 (29.9)	61 (30.7)	99 (52.9)	<0.0001
Diabetes	14 (18.2)	37 (18.6)	52 (27.8)	0.060
Hyperlipidemia	29 (37.7)	92 (46.2)	136 (72.7)	<0.001
Embolism	24 (31.2)	46 (23.1)	43 (23.0)	0.318
Chronic kidney disease	1 (1.3)	11 (5.5)	16 (8.6)	0.073
Gastrointestinal bleeding	1 (1.3)	5 (2.5)	3 (1.6)	0.734
Location of ventricular thrombus				<0.0001
Left ventricular	58 (75.3)	179 (89.9)	185 (98.9)	—
Right ventricular	12 (15.6)	17 (8.5)	1 (0.5)	—
Biventricular	7 (9.1)	3 (1.5)	1 (0.5)	—
Number of ventricular thrombi				0.004
1	66 (85.7)	180 (90.5)	181 (96.8)	—
≥2	11 (14.3)	19 (9.5)	6 (3.2)	—
Size of ventricular thrombus				
Diameter, mm	22.0 (16.0–30.0)	22.0 (15.0–31.0)	22.0 (16.0–31.0)	0.636
Thickness, mm	15.5 (12.5–21.5)	15.0 (11.0–21.0)	17.0 (11.0–23.5)	0.606
LVEF, % (median (IQR))	31.0 (25.0–45.0)	31.5 (24.0–42.0)	40.0 (33.0–50.0)	<0.0001
D-Dimer, ug/mL [mean ± SD]	1.4 (0.6–2.8)	1.3 (0.5–2.7)	0.7 (0.4–1.5)	0.249
Combined medications				
Parenteral anticoagulants	30 (39.0)	150 (75.4)	122 (65.2)	<0.0001
Antiplatelet therapy	26 (33.8)	81 (40.7)	—	<0.0001

^†^Other diagnoses included peripartum cardiomyopathy, myocarditis, arrhythmogenic right ventricular cardiomyopathy, hypertensive heart disease, and noncompaction of ventricular myocardium. VT: ventricular thrombus; *N*: number of patients; SD: standard deviation; IQR: interquartile range; NOACs: non-vitamin K antagonist oral anticoagulants; VKAs: vitamin K antagonists; BMI: body mass index; ICM: ischemic cardiomyopathy; DCM: dilated cardiomyopathy; HCM: hypertrophic cardiomyopathy; LVEF: left ventricular ejection fraction.

**Table 2:** Outcomes of thrombus resolution in patients with an imaging follow-up at different time points (*N* (%)).

Thrombus resolution	NOACs (*n* = 53)	VKAs (*n* = 92)	Antiplatelet therapy (*n* = 67)	*p* value^*∗*^
6 weeks	27 (50.94)	20 (21.74)	16 (23.88)	0.0005
12 weeks	37 (69.81)	38 (41.3)	31 (46.27)	0.0032
6 months	44 (83.02)	63 (68.48)	44 (65.67)	0.0844
12 months	46 (86.79)	71 (77.17)	55 (82.09)	0.3515

Note: a total of 251 patients were lost to the imaging follow-up: 246 (98%) patients had the phone review without the image reports, and five other patients were dead not until the first visit follow-up at 6 weeks, three (1.2%) patients in the VKAs group died from heart failure during the initial hospitalization, and two (0.8%) patients in the APT group due to the aggravating multiorgan diseases, separately occurring at 25 days later after discharge. ^*∗*^Calculated by Chi-square test. VT: ventricular thrombus; *N*: number of patients; NOACs: non-vitamin K antagonist oral anticoagulants; VKAs: vitamin K antagonists; APT: antiplatelet therapy.

**Table 3:** Main results of Cox proportional hazards regression analysis^†^.

Variable	Univariable		Multivariable^‡^	
	HR (95% CI)	*p* value	HR (95% CI)	*p* value
Treatments				
NOACs versus VKAs	2.28 (1.57, 3.31)	<0.001	2.13 (1.41, 3.22)	<0.001
NOACs versus antiplatelet therapy	2.92 (1.97, 4.33)	<0.001	2.55 (1.53, 4.27)	<0.001
VKAs versus antiplatelet therapy	1.28 (0.90, 1.82)	0.167	1.20 (0.76, 1.90)	0.440
Demography				
Age	0.98 (0.97, 0.99)	<0.0001	—	—
Male (versus women)	0.69 (0.48, 0.98)	0.036	—	—
Presenting diagnosis				
ICM (versus DCM)	0.69 (0.45, 1.04)	0.098	—	—
ICM (versus HCM)	1.42 (0.44, 4.54)	0.558	—	—
ICM (versus others^§^)	0.66 (0.42, 1.01)	0.057	—	—
DCM (versus HCM)	2.07 (0.64, 6.69)	0.226	—	—
Prior medical history				
Coronary artery disease	0.61 (0.45, 0.82)	0.001	—	—
Atrial fibrillation	0.80 (0.46, 1.39)	0.431	—	—
Heart failure	1.50 (1.11, 2.02)	0.008	—	—
Hypertension	0.82 (0.60, 1.12)	0.217	—	—
Hyperlipidemia	0.76 (0.57, 1.03)	0.076	—	—
Locations of ventricular thrombus			—	—
Left ventricular (versus right ventricular)	1.14 (0.60, 2.16)	0.690	—	—
Number of ventricular thrombi			—	—
1 (versus ≥ 2)	0.93 (0.54, 1.60)	0.790	—	—
LVEF	0.99 (0.98, 0.999)	0.032	—	—
D-Dimer	0.94 (0.88, 0.999)	0.047	—	—
Combined medications			—	—
Parenteral anticoagulants	0.79 (0.58, 1.07)	0.132	—	—
Antiplatelet therapy	0.82 (0.57, 1.19)	0.300	—	—

^†^In the Cox proportional hazards regression analysis, the end point was thrombus resolution in patients with VT within 12 months' follow-up. ^‡^Adjusted for age, sex, presenting diagnosis, coronary artery disease, heart failure, LVEF, and D-Dimer. ^§^Other diagnoses included peripartum cardiomyopathy, myocarditis, arrhythmogenic right ventricular cardiomyopathy, hypertensive heart disease, and noncompaction of ventricular myocardium. VT: ventricular thrombus; *N*: number of patients; NOACs: non-vitamin K antagonist oral anticoagulants; VKAs: vitamin K antagonists; ICM: ischemic cardiomyopathy; DCM: dilated cardiomyopathy; HCM: hypertrophic cardiomyopathy; LVEF: left ventricular ejection fraction; HR: hazard ratio, CI: confidence interval.

**Table 4:** The secondary outcomes of VT patients within 12 months' follow-up (*N* (%)).

	NOACs (*n* = 77)	VKAs (*n* = 199)	Antiplatelet therapy (*n* = 187)	Total (*n* = 463)	*p* value^*∗*^
Bleeding	1 (1.3)	12 (6.0)	12 (6.4)	25 (5.4)	0.216
Thromboembolism	0 (0)	1 (0.5)	1 (0.5)	2 (0.4)	1.000
All-cause death	0 (0)	5 (2.5)	2 (1.1)	7 (1.5)	0.392

^*∗*^Calculated by Fisher's exact test. VT: ventricular thrombus; *N*: number of patients; NOACs: non-vitamin K antagonist oral anticoagulants; VKAs: vitamin K antagonists.

## Data Availability

The data used to support the findings of this study are available from the corresponding author upon request.

## Supplementary Materials

Supplementary Materials Table S1: the number and the proportion of patients with ventricular thrombus. VT: ventricular thrombus; *N*: number of patients. Table S2: baseline characteristics of patients with or without the imaging follow-up [*N* (%)]. ^*∗*^Other diagnoses included peripartum cardiomyopathy, myocarditis, arrhythmogenic right ventricular cardiomyopathy, hypertensive heart disease, and noncompaction of ventricular myocardium. VT: ventricular thrombus; *N*: number of patients; SD: standard deviation; IQR: interquartile range; BMI: body mass index; ICM: ischemic cardiomyopathy; DCM: dilated cardiomyopathy; HCM: hypertrophic cardiomyopathy; LVEF: left ventricular ejection fraction. Table S3: baseline characteristics of patients who had the imaging follow-up [*N* (%)]. ^*∗*^Other diagnoses included peripartum cardiomyopathy, myocarditis, arrhythmogenic right ventricular cardiomyopathy, hypertensive heart disease, and noncompaction of ventricular myocardium. VT: ventricular thrombus; *N*: number of patients; SD: standard deviation; IQR: interquartile range; NOACs: non-vitamin K antagonist oral anticoagulants; VKAs: vitamin K antagonists; BMI: body mass index; ICM: ischemic cardiomyopathy; DCM: dilated cardiomyopathy; HCM: hypertrophic cardiomyopathy; LVEF: left ventricular ejection fraction. Table S4: subgroup analyses on the primary and the secondary outcomes within follow-up periods [*N* (%)]. *N*: number of patients; NOACs: non-vitamin K antagonist oral anticoagulants; VKAs: vitamin K antagonists. Table S5: other results of Cox proportional hazards regression analysis^*∗*^. ^*∗*^In the Cox proportional hazards regression analysis, the end point was thrombus resolution in patients with VT within 12 months' follow-up. ^*∗∗*^Variables were from those nonsignificant variables in the analysis of variance or Kruskal–Wallis H test for continuous variables and the Pearson chi-squared test or Fisher's exact test for categorical data, shown in Table 1. ^*∗∗∗*^Other diagnoses included peripartum cardiomyopathy, myocarditis, arrhythmogenic right ventricular cardiomyopathy, hypertensive heart disease, and noncompaction of ventricular myocardium. VT: ventricular thrombus; *N*: number of patients; NOACs: non-vitamin K antagonist oral anticoagulants; VKAs: vitamin K antagonists; VA: ventricular aneurysm; ICM: ischemic cardiomyopathy; DCM: dilated cardiomyopathy; HCM: hypertrophic cardiomyopathy; LVEF: left ventricular ejection fraction; HR: hazard ratio, CI: confidence interval. Table S6: literature reviewed [*N* (%)]. *N*: number of patients; NOACs: non-vitamin K antagonist oral anticoagulants; VKAs: vitamin K antagonists.
