# Supplementary material for: Different Oral Antithrombotic Therapy for the Treatment of Ventricular Thrombus: An Observational Study from 2010 to 2019
Source: Int J Clin Pract. 2022 Feb 24;2022:7400860. doi: 10.1155/2022/7400860 (PMC9159124; doi:10.1155/2022/7400860)
Supplement: Supplementary Materials — Table S1: the number and the proportion of patients with ventricular thrombus. VT: ventricular thrombus; N: number of patients. Table S2: baseline characteristics of patients with or without the imaging follow-up [N (%)]. ∗Other diagnoses included peripartum cardiomyopathy, myocarditis, arrhythmogenic right ventricular cardiomyopathy, hypertensive heart disease, and noncompaction of ventricular myocardium. VT: ventricular thrombus; N: number of patients; SD: standard deviation; IQR: interquartile range; BMI: body mass index; ICM: ischemic cardiomyopathy; DCM: dilated cardiomyopathy; HCM: hypertrophic cardiomyopathy; LVEF: left ventricular ejection fraction. Table S3: baseline characteristics of patients who had the imaging follow-up [N (%)]. ∗Other diagnoses included peripartum cardiomyopathy, myocarditis, arrhythmogenic right ventricular cardiomyopathy, hypertensive heart disease, and noncompaction of ventricular myocardium. VT: ventricular thrombus; N: number of patients; SD: standard deviation; IQR: interquartile range; NOACs: non-vitamin K antagonist oral anticoagulants; VKAs: vitamin K antagonists; BMI: body mass index; ICM: ischemic cardiomyopathy; DCM: dilated cardiomyopathy; HCM: hypertrophic cardiomyopathy; LVEF: left ventricular ejection fraction. Table S4: subgroup analyses on the primary and the secondary outcomes within follow-up periods [N (%)]. N: number of patients; NOACs: non-vitamin K antagonist oral anticoagulants; VKAs: vitamin K antagonists. Table S5: other results of Cox proportional hazards regression analysis∗. ∗In the Cox proportional hazards regression analysis, the end point was thrombus resolution in patients with VT within 12 months' follow-up. ∗∗Variables were from those nonsignificant variables in the analysis of variance or Kruskal–Wallis H test for continuous variables and the Pearson chi-squared test or Fisher's exact test for categorical data, shown in Table 1. ∗∗∗Other diagnoses included peripartum cardiomyopathy, myocarditis, arr [file 7400860.f1.docx]

**Different Oral Antithrombotic Therapy for the Treatment of Ventricular Thrombus: An Observational Study From 2010-2019**

Qing Yang^1*^, Xinyue Lang^1,2*^, Xin Quan^1^, Zebin Gong^1^, Yan Liang^1#^

1. Fuwai Hospital, Chinese Academy of Medical Sciences & Peking Union Medical College, Beijing 100037, China
2. Medical Research & Biometrics Center, National Center for Cardiovascular Diseases, Chinese Academy of Medical Sciences, Beijing 102300, China

*These authors contributed to the work equally and should be regarded as co-first authors.

[Table Legends 2](#_Toc91790393)

[Supplemental Table 3](#_Toc91790394)

Table Legends

**Table S1**. The number and the proportion of patients with ventricular thrombus

**Abbreviations:** VT: ventricular thrombus; N: numbers of patients.

**Table S2.** Baseline characteristics of patients with or without the imaging follow-up [N (%)]

* Other diagnoses included peripartum cardiomyopathy, myocarditis, arrhythmogenic right ventricular cardiomyopathy, hypertensive heart disease and noncompaction of ventricular myocardium.

**Abbreviations:** VT: ventricular thrombus; N: numbers of patients; SD: standard deviation; IQR: interquartile range; BMI: body mass index; ICM: ischemic cardiomyopathy; DCM: dilated cardiomyopathy; HCM: hypertrophic cardiomyopathy; LVEF: left ventricular ejection fraction.

**Table S3.** Baseline characteristics of patients who had the imaging follow-up [N (%)]

* Other diagnoses included peripartum cardiomyopathy, myocarditis, arrhythmogenic right ventricular cardiomyopathy, hypertensive heart disease and noncompaction of ventricular myocardium.

**Abbreviations:** VT: ventricular thrombus; N: numbers of patients; SD: standard deviation; IQR: interquartile range; NOACs: non-vitamin K antagonist oral anticoagulants; VKAs: vitamin K antagonists; BMI: body mass index; ICM: ischemic cardiomyopathy; DCM: dilated cardiomyopathy; HCM: hypertrophic cardiomyopathy; LVEF: left ventricular ejection fraction.

**Table S4.** Subgroup analyses on the primary and the secondary outcome within follow-up periods [N (%)]

**Abbreviations:** N: numbers of patients; NOACs: non-vitamin K antagonist oral anticoagulants; VKAs: vitamin K antagonists.

**Table S5.** Other results of Cox proportional hazards regression analysis*

*In the Cox proportional hazards regression analysis, the end point was thrombus resolution in patients with VT within 12 months follow-up.

**Variables were from those not-significant variables in the analysis of variance or Kruskal-Wallis H test for continuous variables and the Pearson chi-squared test or Fisher’s exact test for categorical data, shown in Table 1.

*** Other diagnoses included peripartum cardiomyopathy, myocarditis, arrhythmogenic right ventricular cardiomyopathy, hypertensive heart disease and noncompaction of ventricular myocardium.

**Abbreviations:** VT: ventricular thrombus; N: numbers of patients; NOACs: non-vitamin K antagonist oral anticoagulants; VKAs: vitamin K antagonists; VA: ventricular aneurysm; ICM: ischemic cardiomyopathy; DCM: dilated cardiomyopathy; HCM: hypertrophic cardiomyopathy; LVEF: left ventricular ejection fraction; HR: hazard ratio, CI: confidence interval.

**Table S6.** Literature reviewed [N (%)]

**Abbreviations:** N: numbers of patients; NOACs: non-vitamin K antagonist oral anticoagulants; VKAs: vitamin K antagonists.

Supplemental Table

**Table S1.** The number and the proportion of patients with ventricular thrombus

| **Year** | **Discharged patients, N** | **Patients with VT, N** | **Proportion of VT, %** |
| --- | --- | --- | --- |
| 2010 | 33,618 | 49 | 0.14% |
| 2011 | 41,519 | 67 | 0.16% |
| 2012 | 46,004 | 77 | 0.17% |
| 2013 | 48,208 | 90 | 0.18% |
| 2014 | 52,017 | 93 | 0.18% |
| 2015 | 56,938 | 27 | 0.05% |
| 2016 | 63,562 | 34 | 0.05% |
| 2017 | 66,830 | 51 | 0.07% |
| 2018 | 69,708 | 87 | 0.12% |
| 2019 | 74,519 | 35 | 0.05% |

**Abbreviations:** VT: ventricular thrombus; N: numbers of patients.

**Table S2.** Baseline characteristics of patients with or without the imaging follow-up [N (%)]

|  | **Patients with the imaging follow-up (N=212)** | **Patients without the imaging follow-up (N=251)** | **p value** |
| --- | --- | --- | --- |
| **Age, y [Mean ± SD]** | 49.7±15.5 | 53.7±14.7 | 0.004 |
| **Male** | 163 (76.9) | 212 (84.5) | 0.038 |
| **BMI, kg/m^2^ [Median (IQR)]** | 24.3 (22.2-26.9) | 24.2 (21.8-26.6) | 0.594 |
| **Presenting diagnosis** |  |  | 0.043 |
| ICM | 58 (27.4) | 80 (31.9) | - |
| Ventricular aneurysm | 66 (31.1) | 98 (39.0) | - |
| DCM | 42 (19.8) | 39 (15.5) | - |
| HCM | 4 (1.9) | 6 (2.4) | - |
| Others* | 42 (19.8) | 28 (11.2) | - |
| **Prior medical history** |  |  |  |
| Coronary artery disease | 123 (58.0) | 179 (71.3) | 0.003 |
| Atrial fibrillation | 15 (7.1) | 28 (11.2) | 0.132 |
| Heart failure | 100 (47.2) | 93 (37.1) | 0.028 |
| Hypertension | 74 (34.9) | 109 (43.4) | 0.062 |
| Diabetes | 39 (18.4) | 64 (25.5) | 0.067 |
| Hyperlipidemia | 111 (52.4) | 146 (58.2) | 0.210 |
| Embolism | 49 (23.1) | 64 (25.5) | 0.552 |
| Chronic kidney disease | 11 (5.2) | 17 (6.8) | 0.476 |
| Gastrointestinal bleeding | 5 (2.4) | 4 (1.6) | 0.553 |
| **Location of ventricular thrombus** |  |  | 0.473 |
| Left ventricular | 192 (90.6) | 230 (91.6) | - |
| Right ventricular | 13 (6.1) | 17 (6.8) | - |
| Biventricular | 7 (3.3) | 4 (1.6) | - |
| **Number of ventricular thrombus** |  |  | 0.605 |
| 1 | 197 (92.9) | 230 (91.6) | - |
| ≥2 | 15 (7.1) | 21 (8.4) | - |
| **Size of ventricular thrombus** |  |  |  |
| Diameter, mm | 23.0 (14.0-32.5) | 21.0 (16.0-29.0) | 0.943 |
| Thickness, mm | 15.0 (11.0-21.0) | 17.0 (12.0-22.0) | 0.231 |
| **LVEF, % [Median (IQR)]** | 35.0 (26.0-45.0) | 38.0 (28.0-48.0) | 0.082 |
| **D-Dimer, ug/mL [Mean ± SD]** | 1.0 (0.4-1.9) | 1.2 (0.4-2.7) | 0.064 |
| **Combined medications** |  |  |  |
| Parenteral anticoagulants | 136 (64.2) | 166 (66.1) | 0.655 |
| Antiplatelet therapy | 47 (22.2) | 60 (23.9) | 0.659 |

* Other diagnoses included peripartum cardiomyopathy, myocarditis, arrhythmogenic right ventricular cardiomyopathy, hypertensive heart disease and noncompaction of ventricular myocardium.

**Abbreviations:** VT: ventricular thrombus; N: numbers of patients; SD: standard deviation; IQR: interquartile range; BMI: body mass index; ICM: ischemic cardiomyopathy; DCM: dilated cardiomyopathy; HCM: hypertrophic cardiomyopathy; LVEF: left ventricular ejection fraction.

**Table S3.** Baseline characteristics of patients who had the imaging follow-up [N (%)]

|  | **NOACs**  **(N=53)** | **VKAs**  **(N=92)** | **Antiplatelet therapy(N=67)** | **p value** |
| --- | --- | --- | --- | --- |
| **Age, y [Mean ± SD]** | 42.7±16.4 | 48.9±15.6 | 56.3±11.5 | <0.0001 |
| **Male** | 37 (69.8) | 72 (78.3) | 54 (80.6) | 0.3482 |
| **BMI, kg/m^2^ [Median (IQR)]** | 23.9 (22.3-26.9) | 24.2 (21.8-27.2) | 24.8 (22.9-26.6) | 0.6596 |
| **Presenting diagnosis** |  |  |  | <0.0001 |
| ICM | 9 (17.0) | 20 (21.7) | 29 (43.3) | - |
| Ventricular aneurysm | 6 (11.3) | 22 (23.9) | 38 (56.7) | - |
| DCM | 16 (30.2) | 26 (28.3) | 0 (0.0) | - |
| HCM | 1 (1.9) | 3 (3.3) | 0 (0.0) | - |
| Others* | 21 (39.6) | 21 (22.8) | 0 (0.0) | - |
| **Prior medical history** |  |  |  |  |
| Coronary artery disease | 17 (32.1) | 44 (47.8) | 62 (92.5) | <0.0001 |
| Atrial fibrillation | 4 (7.5) | 8 (8.7) | 3 (4.5) | 0.5848 |
| Heart failure | 33 (62.3) | 56 (60.9) | 11 (16.4) | <0.0001 |
| Hypertension | 12 (22.6) | 29 (31.5) | 33 (49.3) | 0.0066 |
| Diabetes | 5 (9.4) | 15 (16.3) | 19 (28.4) | 0.0231 |
| Hyperlipidemia | 16 (30.2) | 44 (47.8) | 51 (76.1) | <0.001 |
| Embolism | 16 (30.2) | 24 (26.1) | 9 (13.4) | 0.0644 |
| Chronic kidney disease | 1 (1.9) | 5 (5.4) | 5 (7.5) | 0.3886 |
| Gastrointestinal bleeding | 1 (1.9) | 4 (4.3) | 0 (0.0) | 0.1969 |
| **Location of ventricular thrombus** |  |  |  | 0.0009 |
| Left ventricular | 41 (77.4) | 84 (91.3) | 67 (100) | - |
| Right ventricular | 7 (13.2) | 6 (6.5) | 0 (0.0) | - |
| Biventricular | 5 (9.4) | 2 (2.2) | 0 (0.0) | - |
| **Number of ventricular thrombus** |  |  |  | 0.0057 |
| 1 | 45 (84.9) | 85 (92.4) | 67 (100) | - |
| ≥2 | 8 (15.1) | 7 (7.6) | 0 (0.0) | - |
| **Size of ventricular thrombus** |  |  |  |  |
| Diameter, mm | 23.0 (17.0-30.0) | 22.0 (12.0-31.0) | 27.5 (18.0-34.0) | 0.6485 |
| Thickness, mm | 16.0 (13.0-22.0) | 14.0 (10.0-21.0) | 15.5 (11.0-21.5) | 0.8430 |
| **LVEF, % [Median (IQR)]** | 30.0 (25.0-45.0) | 31.0 (24.0-40.0) | 40.0 (32.0-46.0) | <0.0001 |
| **D-Dimer, ug/mL [Mean ± SD]** | 1.4 (0.5-2.6) | 1.0 (0.4-2.0) | 0.6 (0.3-1.4) | 0.2105 |
| **Combined medications** |  |  |  |  |
| Parenteral anticoagulants | 21 (39.6) | 65 (70.7) | 50 (74.6) | <0.0001 |
| Antiplatelet therapy | 14 (26.4) | 33 (35.9) | 0 (0.0) | <0.0001 |

* Other diagnoses included peripartum cardiomyopathy, myocarditis, arrhythmogenic right ventricular cardiomyopathy, hypertensive heart disease and noncompaction of ventricular myocardium.

**Abbreviations:** VT: ventricular thrombus; N: numbers of patients; SD: standard deviation; IQR: interquartile range; NOACs: non-vitamin K antagonist oral anticoagulants; VKAs: vitamin K antagonists; BMI: body mass index; ICM: ischemic cardiomyopathy; DCM: dilated cardiomyopathy; HCM: hypertrophic cardiomyopathy; LVEF: left ventricular ejection fraction.

**Table S4.** Subgroup analyses on the primary and the secondary outcome within follow-up periods [N (%)]

| **Groups** | **Thrombus resolution** | **Bleeding** | **Thromboembolism** | **All-cause death** |
| --- | --- | --- | --- | --- |
| **NOACs** |  |  |  |  |
| Rivaroxaban | 43/48(89.6) | 1/72(1.4) | 0/72 | 0/72 |
| Dabigatran etexilate | 2/4 | 0/4 | 0/4 | 0/4 |
| Apixaban | 1/1 | 0/1 | 0/1 | 0/1 |
| **VKAs** |  |  |  |  |
| Warfarin | 71/92(77.2) | 12/199(6.0) | 1/199(0.5) | 5/199(2.5) |
| **Antiplatelet therapy** |  |  |  |  |
| Aspirin/Clopidogrel | 7/10 | 3/32(9.4) | 1/32(3.1) | 1/32(3.1) |
| Aspirin + Clopidogrel or Ticagrelor | 48/57(84.2) | 9/155(5.8) | 0/155 | 1/155(0.6) |

**Abbreviations:** N: numbers of patients; NOACs: non-vitamin K antagonist oral anticoagulants; VKAs: vitamin K antagonists.

**Table S5.** Other results of Cox proportional hazards regression analysis*

| **Variable**** | **Univariable** | |
| --- | --- | --- |
|  | **HR (95% CI)** | **p value** |
| **Demography** |  |  |
| BMI | 1 (0.98, 1.01) | 0.706 |
| **Presenting diagnosis** |  |  |
| ICM (vs VA) | 1.4 (0.94, 2.09) | 0.098 |
| VA (vs DCM) | 0.49 (0.32, 0.75) | <0.001 |
| VA (vs HCM) | 1.01 (0.31, 3.25) | 0.987 |
| VA (vs Others***) | 0.47 (0.3, 0.73) | <0.001 |
| DCM (vs Others) | 0.96 (0.6, 1.51) | 0.848 |
| HCM (vs Others) | 0.46 (0.14, 1.51) | 0.201 |
| **Prior medical history** |  |  |
| Diabetes | 0.78 (0.53, 1.14) | 0.191 |
| Embolism | 0.8 (0.55, 1.15) | 0.225 |
| Chronic kidney disease | 0.76 (0.37, 1.55) | 0.451 |
| Gastrointestinal bleeding | 0.81 (0.26, 2.54) | 0.721 |
| **Locations of ventricular thrombus** |  |  |
| Left ventricular (vs right ventricular) | 1.14 (0.6, 2.16) | 0.690 |
| **Size of ventricular thrombus** |  |  |
| Diameter | 0.99 (0.97, 1.01) | 0.032 |
| Thickness | 0.98 (0.96, 1) | 0.047 |

*In the Cox proportional hazards regression analysis, the end point was thrombus resolution in patients with VT within 12 months follow-up.

**Variables were from those not-significant variables in the analysis of variance or Kruskal-Wallis H test for continuous variables and the Pearson chi-squared test or Fisher’s exact test for categorical data, shown in Table 1.

*** Other diagnoses included peripartum cardiomyopathy, myocarditis, arrhythmogenic right ventricular cardiomyopathy, hypertensive heart disease and noncompaction of ventricular myocardium.

**Abbreviations:** VT: ventricular thrombus; N: numbers of patients; NOACs: non-vitamin K antagonist oral anticoagulants; VKAs: vitamin K antagonists; VA: ventricular aneurysm; ICM: ischemic cardiomyopathy; DCM: dilated cardiomyopathy; HCM: hypertrophic cardiomyopathy; LVEF: left ventricular ejection fraction; HR: hazard ratio, CI: confidence interval.

**Table S6.** Literature reviewed [N (%)]

| **Study** | **Comparison** | **Sample size** | **Thrombus resolution** | **Bleeding** | **Stroke or systemic embolism** | **All-cause death** |
| --- | --- | --- | --- | --- | --- | --- |
| **Yao et al,**  **2021** | NOACs | 42 | 29(69) | 0 | - | - |
|  | VKA | 58 | 37(64) | 0 | 2(3) | - |
| **Iskaros et al,**  **2021** | NOACs | 32 | 27(84) | 2(7) | 5(17) | - |
|  | VKA | 45 | 34(75) | 2(5) | 11(26) | - |
| **Zhang et al,**  **2021** | NOACs | 33 | 26(78.8) | 0 | 1(3) | 1(3) |
|  | VKA | 31 | 23(74.2) | 1(1.6) | 4(12.9) | 4(12.9) |
| **Mihm et al,**  **2021** | NOACs | 33 | 14/24(58.3) | 5(15) | 3(9.1) | 4(12.1) |
|  | VKA | 75 | 26/40(65) | 2(2.7) | 4(5.3) | 6(8) |
| **Alcalai et al,**  **2021** | NOACs | 18 | 16(94.1) | 0 | 0 | 1(5.56) |
|  | VKA | 17 | 14(93.3) | 2(11.76) | 1(5.88) | 0 |
| **Albabtain et al, 2021** | NOACs | 28 | 20(71.4) | 2(7.14) | 2(7.14) | 2(7.14) |
|  | VKA | 35 | 24(68.6) | 1(2.86) | 1(2.86) | 3(8.57) |
| **Varwani et al, 2021** | NOACs | 58 | 22(61.1) | 3(5.2) | 1(1.7) | 0 |
|  | VKA | 34 | 16(64) | 2(5.9) | 1(2.9) | 0 |
| **Jones et al,**  **2020** | NOACs | 41 | 39(95) | 0 | 1(2.4) | 0 |
|  | VKA | 60 | 51(85) | 4(6.7) | 3(5) | 0 |
| **Daher et al,**  **2020** | NOACs | 17 | 12(71) | - | 2(12) | 0 |
|  | VKA | 42 | 30(71) | - | 4(9) | 0 |
| **Yunis et al,**  **2020** | NOACs | 64 | 64(100) | 26(40) | 3(5) | 12(19) |
|  | VKA | 200 | 194(97) | 70(35) | 16(8) | 42(21) |
| **Ali et al,**  **2020** | NOACs | 32 | 18(53) | 2(6) | - | - |
|  | VKA | 60 | 37(63) | 15(27) | 2(3) | - |
| **Iqbal et al,**  **2020** | NOACs | 22 | 13(65) | 0 | 0 | 3(14) |
|  | VKA | 62 | 42(76) | 3(5) | 2(3) | 6(10) |
| **Robinson et al,**  **2020** | NOACs | 121 | 56(46) | 8(7) | 17(14) | 15(12) |
|  | VKA | 236 | 131(55) | 19(8) | 14(6) | 33(14) |
| **Willeford et al,**  **2020** | NOACs | 22 | 13(59) | 0 | 1(4) | - |
|  | VKA | 129 | 63(49) | 8(6) | 5(4) | - |
| **Bass et al,**  **2020** | NOACs | 180 | - | 60(33) | 20(11) | - |
|  | VKA | 769 | - | 235(31) | 60(8) | - |
| **Guddeti et al,**  **2020** | NOACs | 19 | 15(80) | 1(5) | 0 | 0 |
|  | VKA | 80 | 65(81) | 4(5) | 2(2) | 0 |
| **Gama et al,**  **2019** | NOACs | 13 | 11(91) | - | - | - |
|  | VKA | 53 | 31(70) | - | - | - |
| **Yan et al,**  **2019** | NOACs | 11 | 7(64) | 0 | 0 | 0 |
|  | VKA | 37 | 19(51) | 2(5) | 3(8) | 1(3) |
| **Alizadeh et al,**  **2019** | NOACs | 38 | 28(75) | 0 | - | - |
|  | VKA | 60 | 32(53) | 3(5) | - | - |
| **McCarthy et al, 2019** | NOACs | 4 | 4(100) | - | - | - |
|  | VKA | 94 | 71(75) | - | - | - |
| **Robinson et al,**  **2018** | NOACs | 35 | - | - | 2(88) | - |
|  | VKA | 40 | - | - | 3(78) | - |
| **Chao et al,**  **2018** | NOACs | 56 | 29(52) | 5(9) | 2(4) | 0 |
|  | VKA | 70 | 22(31) | 11(16) | 12(17) | 0 |
| **Li et al,**  **2015** | NOACs | 15 | 13(87) | 0 | 1(7) | 0 |
|  | VKA | 16 | 12(75) | 0 | 1(6) | 0 |

**Abbreviations:** N: numbers of patients; NOACs: non-vitamin K antagonist oral anticoagulants; VKAs: vitamin K antagonists.
